# Exploring the behavioral addiction comorbidities in patients with bipolar disorders – A literature review

**DOI:** 10.1192/j.eurpsy.2026.10802

**Published:** 2026-07-31

**Authors:** O. Vasiliu, A. G. Mangalagiu, B. M. Petrescu, C. A. Cândea, C. Tudor, D. Ungureanu, M. Miclos, C. Florescu, R. E. Bratu-Bizic, M. Dobre, A. F. Fainarea, M. Patrascu, M. Sandru

**Affiliations:** 1 Clinical Neurosciences Department, “Carol Davila” University of Medicine and Pharmacy; 2 Psychiatry Department, “Dr. Carol Davila” University Emergency Central Military Hospital, Bucharest, Romania

## Abstract

**Introduction:**

The rate of substance use disorder (SUDs) in patients with bipolar depression (BD) is reputedly high, with different values reported, that may exceed 40% for alcohol use disorder and 20% for cannabis use disorder. However, the rate of behavioral addictions (BA) in this vulnerable population has been less explored, although the neuropathology of SUD and BA is suggested to share a common background.

**Objectives:**

The objective of this review was to explore the available data in the literature regarding the comorbidities of BD and BA.

**Methods:**

A literature review was conducted in three electronic databases (PubMed, EMBASE, and Clarivate/Web of Science) using the keywords „bipolar disorder,” „behavioral addiction,” „gambling disorder,” „shopping disorder,” „gaming disorder,” and „Internet use disorder” for papers published in English between January 2000 and February 2025.

**Results:**

Based on the results of 35 primary and secondary reports, the prevalence of BA was significantly higher than in the controls (the values in the BD group were more than double and up to six times that in controls for most of the studies). Gambling disorder (GD) seems to be the most frequently reported BA in patients with BD, but the prevalence of this association is debated by different authors, due to the methodological heterogeneity of the reviewed studies. Shopping disorder was also reported frequently in patients with BD, but the need to differentiate this disorder as an independent diagnosis from the tendency to overspend due to manic episodes is challenging. Also, Internet addiction should be screened for in these patients, and a high rate of multiple SUD and BA comorbidity in patients with BD was also reported. Caution is needed in the case of dual, BD and BA diagnoses, because of the potential misattribution of core BD symptoms to BA, especially because the diagnostic criteria for BA are still controversial, except for GD. Therefore, the use of structured methods, like psychometric tools dedicated to BD and BA, in combination with a thorough clinical assessment, is needed.

**Conclusions:**

There is an acute need to screen for BA in patients with BD because this comorbidity has important therapeutic consequences and a potential relevant impact on their evolution and prognosis.

**Disclosure of Interest:**

None Declared

